# Variation in the Genetic Repertoire of Viruses Infecting *Micromonas pusilla* Reflects Horizontal Gene Transfer and Links to Their Environmental Distribution

**DOI:** 10.3390/v9050116

**Published:** 2017-05-19

**Authors:** Jan F. Finke, Danielle M. Winget, Amy M. Chan, Curtis A. Suttle

**Affiliations:** 1 Department of Earth, Ocean and Atmospheric Sciences and Biodiversity Research Centre, The University of British Columbia, Vancouver, BC V6T 1Z4, Canada; jfinke@eos.ubc.ca (J.F.F.); wingetde@ucmail.uc.edu (D.M.W.); chanamym@mail.ubc.ca (A.M.C.); 2 Institute for the Oceans and Fisheries, The University of British Columbia, Vancouver, BC V6T 1Z4, Canada; 3 Department of Microbiology and Immunology, The University of British Columbia, Vancouver, BC V6T 1Z3, Canada; 4 Department of Botany, The University of British Columbia, Vancouver, BC V6T 1Z4, Canada; 5 Integrated Program in Microbial Biodiversity, Canadian Institute for Advanced Research, Toronto, ON M5G 1Z8, Canada

**Keywords:** prasinophytes, *Phycodnaviridae*, core-genome, pan-genome, horizontal gene transfer

## Abstract

Prasinophytes, a group of eukaryotic phytoplankton, has a global distribution and is infected by large double-stranded DNA viruses (prasinoviruses) in the family *Phycodnaviridae*. This study examines the genetic repertoire, phylogeny, and environmental distribution of phycodnaviruses infecting *Micromonas pusilla*, other prasinophytes and chlorophytes. Based on comparisons among the genomes of viruses infecting *M. pusilla* and other phycodnaviruses, as well as the genome from a host isolate of *M. pusilla*, viruses infecting *M. pusilla* (MpVs) share a limited set of core genes, but vary strongly in their flexible pan-genome that includes numerous metabolic genes, such as those associated with amino acid synthesis and sugar manipulation. Surprisingly, few of these presumably host-derived genes are shared with *M. pusilla*, but rather have their closest non-viral homologue in bacteria and other eukaryotes, indicating horizontal gene transfer. A comparative analysis of full-length DNA polymerase (DNApol) genes from prasinoviruses with their overall gene content, demonstrated that the phylogeny of DNApol gene fragments reflects the gene content of the viruses; hence, environmental DNApol gene sequences from prasinoviruses can be used to infer their overall genetic repertoire. Thus, the distribution of virus ecotypes across environmental samples based on DNApol sequences implies substantial underlying differences in gene content that reflect local environmental conditions. Moreover, the high diversity observed in the genetic repertoire of prasinoviruses has been driven by horizontal gene transfer throughout their evolutionary history, resulting in a broad suite of functional capabilities and a high diversity of prasinovirus ecotypes.

## 1. Introduction

Prasinophytes are a divergent group of marine eukaryotic phytoplankton within the division Chlorophyta [1]. They have a global distribution and are a major component of coastal and oceanic communities, and include the prominent genera *Micromonas*, *Ostreococcus*, and *Bathyococcus*. They, arguably, constitute the second-most abundant group of phytoplankton, after cyanobacteria, with a high importance in primary production [2,3].

Prasinophytes are infected by large icosahedral viruses with double-stranded DNA genomes [4] that belong to the genera *Prasinovirus*. These viruses and the related *Chloroviruses* are members of the family *Phycodnaviridae* [5] which share properties with other nucleocytoplasmic large DNA viruses (NCLDV) [6]. NCLDVs include viruses infecting amoeba and mammals, but phycodnaviruses are only known to infect algae. While NCLDV genomes range from 100 kb to >1 Mb in size [6], characterized phycodnavirus genomes range from 150 to 560 kb [7]. These viruses can be significant agents of mortality and influence nutrient fluxes, host diversity, or act in horizontal gene transfer [8,9].

In recent years several prasinovirus genomes have been sequenced. The genome of *Micromonas pusilla* virus MpV1 is 184,095 bp; genomes of *Ostreococcus tauri* virus OtV5 and *O. lucimarinus* virus OlV2 are 186,234 and 196,300 bp, respectively, and those of the *Bathyococcus prasinos* viruses BpV2 and BpV1 are 187,069 are 198,519 bp, respectively [10,11,12]. Generally, prasinoviruses are similar in genome structure and content, and show a high degree of orthology and synteny [12,13].

Despite the similarity in genome architecture among prasinoviruses, they are typically host specific within a species, as shown for viruses infecting *Ostreococcus* spp. [11]. As well, viruses infecting *M. pusilla* do not infect *Ostreococcus* spp.; yet, they infect *M. pusilla* strains from different origins [14]. However, some viruses infect and potentially incorporate genes across genera [15]; thus, prasinoviruses have the potential to acquire genes from different host genera. Consequently, the genome of prasinoviruses is comprised of a small set of core genes and a larger flexible genome of various origins.

Although the functions of most prasinovirus genes are unknown [16], a set of core genes that are typically clustered together and essential for viral replication and structure have been identified [10,11,13]. These core genes include DNA polymerase, DNA topoisomerase, and seven to eight genes encoding capsid proteins. In contrast, the flexible pan-genome comprises many genes of unknown function, but also contains some metabolic genes similar to the auxiliary metabolic genes (AMGs) found in cyanophages [17]. The genomes vary in the transfer RNAs (tRNAs) [12] and K^+^ channels [18] that are encoded. Additionally, *Ostreococcus* viruses possess genes of presumed cellular origin, some with homologues in their hosts. These include genes associated with sugar manipulation (glycosyltransferases), nucleotide modification (ribonucleotide reductase), amino acid synthesis (acetolacetate synthase), phosphate starvation (*phoH*), and many more [13]. Moreau et al. [12] have also shown that there are homologues of cell-derived genes in viruses that infect members of the genera *Bathyococcus*, *Ostreococcus*, and *Micromonas*. Assuming that these genes are expressed during viral replication, the viruses carrying them will be more “fit” under conditions where these genes carry a selective advantage; yet, the distribution of pan-genes among prasinoviruses remains to be studied.

Core genes have been used as targets to investigate the distribution and diversity of specific groups of viruses across environments. Moreover, because these genes are conserved and not laterally transferred, they can be used to build phylogenetic relationships within groups of viruses. Viruses which are most closely related would also be expected to be similar in terms of overall gene content; hence, viruses that are most similar to each other with respect to the phylogeny of their core genes would be expected to share a similar genetic repertoire. As genes encoding DNA polymerase B (DNApol) and the major capsid protein (MCP) have been used extensively to study phycodnavirus diversity, if core gene phylogenies provide insight into virus gene content, this provides an opportunity to infer how the “metabolic potential” of phycodnaviruses varies across environments.

In particular, DNApol sequences have been used to infer diversity and phylogenetic relationships among phycodnaviruses in marine [19,20,21,22] and freshwater [23,24,25] environments. Similarly, MCP has been used as a marker of phycodnavirus diversity [26,27]. Clerissi et al. [28] used full and partial DNApol and MCP gene sequences to phylogenetically compare prasinoviruses and chloroviruses, and showed that full-gene phylogenies for DNApol and MCP were congruent. However, looking at diversity and phylogeny with amplicon sequences is compromised because of the specificity of the primers. For example, the primers typically used for DNApol [21] amplify MpV sequences [23,24], whereas, the primers used for MCP miss them [26]. These differences were highlighted in a freshwater study [29] in which primers for DNApol and MCP favored amplification of prasinovirus and prymnesiovirus sequences, respectively.

Another approach to examine the genetic relatedness among viruses is to build multi-gene phylogenies. This can be done based on selected core genes [11], or by comparing the presence and absence of genes across entire genomes. Gene presence-absence trees provide a rigorous way to examine evolutionary relationships among large DNA viruses [30,31], but the approach is not amenable to comparing viruses based on environmental sequence data. Nonetheless, gene presence-absence trees can be used to construct robust phylogenetic relationships among sequenced virus isolates which, in turn, can serve as a backbone for making predictions about virus gene content from environmental amplicon-based sequencing data. Therefore, a relationship between a phylogeny based on core gene sequences, such as for DNApol, and overall gene content would need to be established. In this way, environmental amplicon data for DNApol can be used to infer the gene content of prasinoviruses in nature. This approach is explored in the present study.

The relationship between virus gene content in prasinoviruses and their environmental distribution is unexplored, but marine virus communities show biogeographic patterns [32,33,34], including viruses infecting *Ostreococcus tauri*, which form distinct communities in contrasting environments [35]. Furthermore, the diversity and composition of prasinovirus communities is influenced by environmental factors, particularly the availability of phosphate [36]. A recent study on cyanophage isolates, which prominently host a range of AMGs, linked their genome similarity with environmental distribution, thus formulating a diversification of viruses into ecotypes [34]. This suggests that the gene content of prasinoviruses may reflect their environmental distribution.

In this study, we contrast the genomes of prasinoviruses infecting *Micromonas pusilla* with those of other phycodnaviruses from a range of hosts and environments, with the goal of describing their genetic composition in the context of their environmental distribution.

## 2. Materials and Methods 

### 2.1. Sequencing and Annotation of *Micromonas pusilla* Viruses

The *Micromonas pusilla* viruses MpV-PL1 and MpV-SP1 were isolated from the mixed layer in the Gulf of Mexico and coastal waters of California (respectively), and propagated on *Micromonas pusilla* (UTEX LB991) [37]. The viruses were purified from 15 mL of lysate by filtration through 0.45 and 0.22 μm pore-size Durapore membrane filters (EMD Millipore Corp., Billerica, MA, USA), ultracentrifugation, and subsequent optiprep gradient centrifugation, as described in Fischer et al. [38]. The DNA was extracted and purified using QIAamp MinElute Virus DNA spin kit (Qiagen, Inc., Valencia, CA, USA) prior to sequencing to 10-fold depth and assembly by the Broad Institute, using the 454 GS FLX platform and Newbler 2.7 (454 Life Sciences, Roche Diagnostics, Basel, Switzerland). Read assembly resulted in two contigs per virus that were mapped in Mauve v2.3.1 [39] to MpV1 as a reference genome. Sequencing gaps were closed by PCR amplification with customized primers (PL1 fwd-GAGGGTGGGCACGTTGGAG, rev-GTCTCTAGGACCCCCACCCT; SP1 fwd-GCTAATGACGAGTTCGGTCG, rev-ACTAAGTAACCGAAACTGTCCCC) to bridge the gaps, cloning of the product and subsequent Sanger sequencing (NAPS, University of British Columbia, Vancouver, BC, Canada). Final genomes were assembled in Geneious 6.0.5 (Biomatters Ltd., Auckland, New Zealand) based on sequence overlap.

To annotate the assembled genomes, open reading frames (ORFs) were called using Artemis v14.0.0 [40] using a minimum ORF length of 65 amino acids (195 nt) with start and stop codons. ORFs were translated into amino-acid sequences using the standard genetic code in three reading frames using Artemis. Putative coding sequences were tested for homology in the nr-database (NCBI) with a protein BLAST (BLAST-P). Annotations were manually selected based on a minimal E-value of E^−10^ and minimum 50% alignment length. tRNAs were determined with tRNAscan-SE v1.21 [41].

### 2.2. Comparing Prasinovirus Genomes and Inferring Viral Phylogeny

Coding sequences (CDS) for MpV-PL1, MpV-SP1, two other sequenced and annotated *Micromonas* viruses MpV1 and MpV-12T, and *M. pusilla* UTEX LB991 were clustered in USEARCH (v6.1.544) [42] based on a 50% pair-wise identity at the amino-acid level. Viral clusters were labeled based on the annotation of MpV-PL1 where applicable. Genome contents were compared based on a cluster presence-absence scheme and Venn diagrams produced in R [43]. Core genes in the *M. pusilla* viruses were defined when a cluster contained CDS from all four genomes, or a CDS could be associated with a cluster based on functional annotation and BLAST-P analysis.

Prasinovirus and chlorovirus genomes were compared phylogenetically. The genomes were clustered using USEARCH v6.1.544, as above, and compared for gene presence or absence. Phylogenetic distances (*Dij*) among genomes were calculated as [*Dij* = −ln(*Sij*/sqrt(Ni*j))], where *Sij*, *Ni*, and *Nj* are the number of shared genes, the number of genes in one genome, and the number of genes in the other genome, respectively [31]. A neighbor-joining tree based on distance was constructed using the APE package [44] in R, and visualized in FigTree v1.4.2 [45]. Bootstrap values for branch support were calculated from 1000 iterations of random gene cluster sub-sampling. Reference DNApol sequences were extracted from the following genomes (accession numbers): MpV1 (NC_014767); MpV-12T (NC_020864); BpV1 (NC_014765); BpV2 (HM004430); OtV1 (NC_013288); OtV2 (NC_014789); OlV1 (NC_014766); OtV5 (NC_010191); OtV6 (JN225873); PBCV1 (NC_000852); and AR158 (NC_009899). Amplicon equivalents of 140 aa length were extracted from the reference sequences and aligned with Clustal Omega v1.2.3 [46]. Phylogenetic distances among the reference viruses were calculated based on maximum-likelihood with the WAG substitution model [47]. The optimal substitution model was selected with ProtTest-v3.4 [48] and distance calculated using RaxML v8.0 [49]. Phylogenetic distances of the gene presence-absence matrix and DNApol were compared with a Mantel test in R.

### 2.3. Assessing the Prevalence of Prasinoviruses in Environmental Samples

Amplicons of DNApol gene fragments were used to infer prasinovirus diversity in environmental samples. Samples of 20 to 72 L of water were taken from the surface at three sites in the Strait of Georgia, Jericho Pier (JP) and Point Atkinson (PA), the Juan de Fuca Strait (JF), and in the surface layer and at 200 m depth, several times per year in Saanich Inlet (SI) (sampling details are available in Supplementary Table S1). JP, PA, and JF samples were sequentially filtered through 47 mm diameter GC50 glass fiber filters (Advantec MFS Inc., Dublin, CA, USA) and HVLP (Millipore, Billerica, MA, USA) membrane filters (~0.45 μm nominal pore-size for each filter). Similarly, Saanich Inlet samples were filtered through 2.7 μm nominal pore size GF/D filters (Whatman, Maidstone, UK) and 0.22 μm pore-size Sterivex filters (EMD Millipore). The remaining particulate matter in each filtrate was then concentrated by tangential flow filtration (TFF) with a 30 kDa molecular-weight cutoff cartridge filter (Prep-Scale, Millipore, Billerica, MA, USA) to make a viral concentrate (VC) that was stored at 4 °C in the dark. For DNA extraction, 14 mL VC subsamples were concentrated by ultracentrifugation for 4 h at 124,000× *g* at 15 °C using a SW40 rotor (Beckman Coulter Life Sciences, Brea, CA, USA), and the pellets resuspended with 500 μL Tris-Ethylenediaminetetraacetic acid (EDTA) (TE) buffer (10 mM-Tris HCl; 1 mM EDTA), pH 8.0) at 4 °C overnight. Samples from Saanich Inlet were pooled into surface layer and deep composites. The viral capsids were lysed with Proteinase K (Invitrogen, Carlsbad, CA, USA) (100 μg mL^−1^) and DNA extracted using phenol-chloroform. Partial DNA polymerase sequences were amplified with AVS1 and AVS2 primers [50], and 500 ng of the PCR products used for library preparation and sequencing with a 454 GS FLX with titanium chemistry (Roche Diagnostics, Basel, CH, Switzerland) at the Broad Institute (Cambridge, MA, USA). Reverse AVS sequences were de-noised using QUIIME v1.4 [51] and chimeras were removed using UCHIME (v4.2.40) [42]. De-noised sequences were translated to amino acids with FragGeneScan v1.16 [52] and dereplicated using USEARCH (v6.1.544). Reads from all environmental samples and reference sequences were pooled and clustered at 97% identity in USEARCH (v8.1). Clusters with only one member were discarded and centroids of the other clusters were aligned with Clustal Omega v1.2.3. Gaps in the alignment were trimmed and a maximum-likelihood tree was built in RaxML 8.0. Environmental reads and reference sequences were parsed using USEARCH (v8.1) at 97% identity. The frequency distribution of parsed environmental reads were rarefied to the lowest number of cumulative reads per sample using the VEGAN package [53] in R. The tree was visualized in iTOL (v3.5) [54].

In situ measurements of temperature (°C) and salinity (Practical Saline Units, PSU) were made with electrodes mounted on a CTD (Seabird, Bellevue, WA, USA) in Saanich Inlet or a YSI probe (YSI, Yellow Springs, OH, USA) in Jericho Pier, Point Atkinson and Juan de Fuca Strait. Additionally, remote sensing data were extracted from Aqua MODIS data (NASA Goddard Space Flight Center, Ocean Ecology Laboratory, Ocean Biology Processing Group) to estimate Chlorophyll *a* concentrations (Chl *a*, mg m^−3^), daytime sea-surface temperature (SST, 4u, °C), photosynthetically active radiation (PAR, μmol photons m^−2^ s^−1^) and particulate organic carbon (POC, mg m^−3^ 443/555) as a rolling 32-day composite pre-dating the sampling period, at a 4-km resolution. Data was processed and mapped in R.

## 3. Results

### 3.1. Origin and Distribution of Genes in *Micromonas* Viruses

The genome sequences of *Micromonas* viruses MpV-PL1 and MpV-SP1 were completed using custom-designed primers. Genomes were analyzed and annotated by BLAST-P analysis of MpV ORFs against the nr-database. This improved earlier annotations, although most ORFs still lack a putative function. The presented study focused on MpV-PL1 and MpV-SP1, and their comparison to the genomes of MpV1 (NC_014767) and MpV-12T (NC_020864). The viruses were isolated on three strains of *M. pusilla* (Table 1) and differed in genome size, the number and average length of their ORFs, GC content, and tRNAs. The genome sizes range from 173,350 bp for MpV-SP1 to 205,622 bp for MpV-12T, which does not correspond to the number or size of ORFs; MpV1 possesses the fewest (244) but, on average, longest ORFs (715 bp), while MpV-PL1 has the most (275), but not the shortest, on average (684 bp). MpV-12T also has the lowest GC content (39.8%), while MpV-PL1 has the highest content (43.3%). Although six tRNAs are common in *Micromonas* viruses, MpV-PL1 lacks Leu-tRNA, while MpV-12T carries two copies of Asn-tRNA.

**Figure 1:**
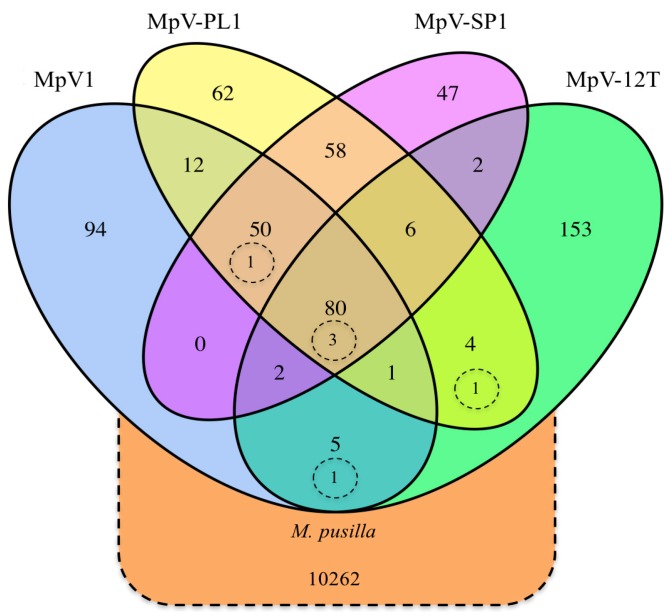
Venn diagram of shared coding sequences (CDS) of four MpVs and *M. pusilla* UTEX LB991, based on clusters by 0.5 amino acid identity. Dashed circles represent host genes shared with viruses.

The cluster analysis of four *Micromonas* viruses and a host (*M. pusilla*, UTEX LB991) genome based on 50% amino acid identity (Figure 1) revealed 80 ORFs shared by all viruses, 140 ORFs are shared in at least two genomes, and 357 ORFs are unique. While MpV1, MpV-PL1, and MpV-SP1 share about half of their ORFs (130), MpV-12T shares only 100 ORFS with any other virus, while 153 ORFS are not shared. In contrast, the genomes of MpV-PL1 and MpV-SP1 have the highest overlap, with 194 ORFs in common. Only six ORFs are shared among the host, *M. pusilla* (UTEX LB991), and the viruses at this similarity level. Three host ORFs occur in all the viruses (ribonucleotide reductase, dUTPase and a cell-division protein), while three others occur in a subset (DNA primase, a heat-shock protein and a hypothetical protein).

Combining the cluster analysis of putative viral genes with an additional BLAST-P analysis against the nr-database revealed a core genome of 119 genes and 327 genes in a pan-genome (Table 2). Core genes include those essential for viral replication and virion structure, such as DNApol, DNA ligase, transcription initiation factor, and seven capsid proteins. Most putative genes are in the flexible pan-genome, including genes which are functionally of cellular origin, such as those involved in carbon metabolism and DNA repair, yet most have no functional annotation. Other putative genes of presumable cellular origin associated with amino acid synthesis, including acetaldehyde dehydrogenase, acetolacetate synthase, and aminotransferase, were found in in MpV1 (12), and MpV-PL1, but are absent in the other *Micromonas* viruses. Heat shock protein 70 was found in MpV-12T and MpV-PL1, and is also shared with *M. pusilla* UTEX LB991. The DNA methylases/methyltranferases are site-specific and differ among the viruses. Moreover, both MpV-PL1 and MpV-SP1 possess a putative host-derived gene for 6-phosphofructokinase, while MpV1, MpV-PL1 and MpV-SP1 share dTDP-d-glucose 4,6-dehydratase. In contrast only MpV-12T carries UDP-glucose 6-dehydrogenase and only MpV-SP1 has two transketolase-related genes. Several other genes are shared among MpV-PL1, MpV-SP1, and MpV1, but not with MpV-12T, which also has the most genes without functional annotation.

**Figure 2:**
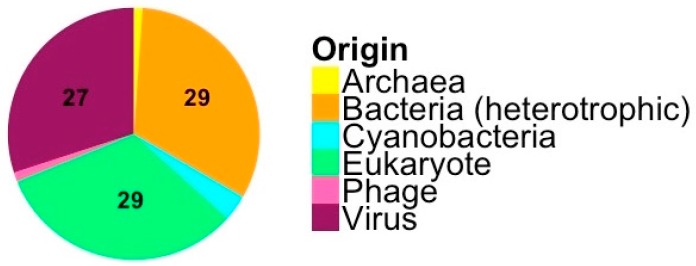
Presumed origin of 90 genes with a functional annotation in the four *M. pusilla* viruses examined in this study. Numbers indicate the number of genes assigned to putative origins. Origin is based on BLAST-P hits against the nr-database.

A BLAST-P analysis against the nr-database of putative coding sequences with a functional annotation revealed that for most core-genes the closest hit is to other virus sequences while, for the flexible genome, the close homologues often are cellular (Figure 2). However, few of the sequences of presumed cellular origin have their closest homologue in *M. pusilla* UTEX LB991, but are rather more similar to sequences in other eukaryotes, heterotrophic bacteria, cyanobacteria, or archaea.

### 3.2. Deriving Similarity in Gene Content from DNApol

**Figure 3:**
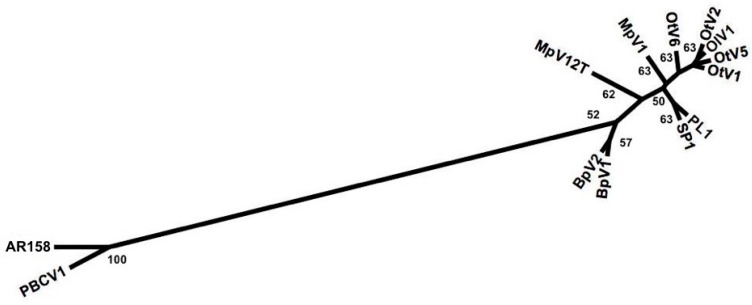
Phylogeny of prasinoviruses infecting the genera *Ostreococcus* (OtV1, OtV2, OtV5, OtV6, OlV1), *Bathyococcus* (BpV1, BpV2), *Micromonas* (MpV1, MpV-12T, MpV-PL1, MpV-SP1), and *Chlorella* (PBCV1, AR158). The neighbor-joining tree is based on the presence and absence of shared putative genes. Bootstrap values are based on 1000 iterations of sub-sampling.

A neighbor-joining phylogenetic analysis of prasinoviruses and chloroviruses based on the presence and absence of putative genes showed the similarity of viruses to each other (Figure 3). The more closely the viruses are related in their gene content, the closer they are on the tree, indicating that the *Chlorella*, *Bathyococcus*, and most *Ostreococcus* viruses form well-defined groups whereas the *Micromonas* viruses form three distinct branches with MpV-PL1 and MpV-SP1 branching together, and MpV1 and MpV-12T being on separate branches.

**Figure 4:**
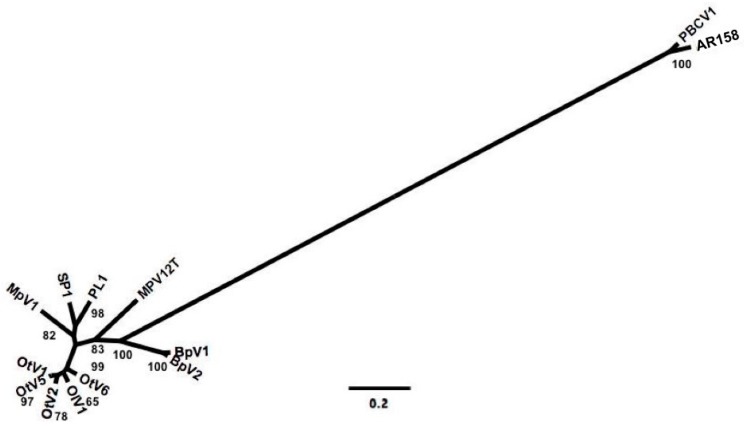
Maximum likelihood tree of prasinoviruses and chloroviruses infecting the genera *Ostreococcus* (OtV1, OtV2, OtV5, OtV6, OlV1), *Bathyococcus* (BpV1, BpV2), *Micromonas* (MpV1, MpV-12T, MpV-PL1, MpV-SP1), and *Chlorella* (PBCV1, AR158). The phylogeny is based on full-length DNA polymerase B (DNApol) sequences, bootstrap values based on 1000 iterations; the scale bar represents the substitution rate.

**Figure 5:**
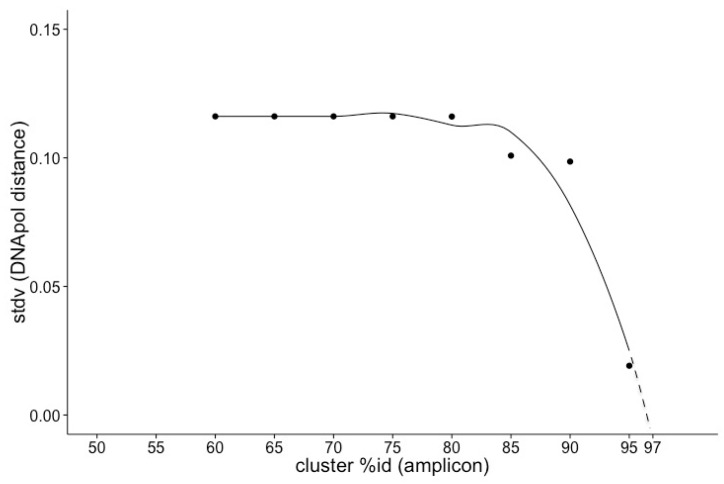
Variation in phylogenetic distance based on DNApol. The standard deviation (stdv) of the pairwise phylogenetic distances of full-length DNApol sequences of reference virus clusters are shown against their corresponding amplicon equivalents at different levels of % amino acid (aa) identity to demonstrate that amplicon sequences clustered at 97% identity are representative of the full-length sequences.

Comparing the phylogenetic relationship among prasinoviruses and chloroviruses from analyses of gene presence and absence, and DNApol sequences drew a congruent picture of the relationship among viruses. This is evident from the topology of phylogenetic trees based on gene presence and absence (Figure 3) and full-length DNApol sequences (Figure 4). Additionally, the phylogenetic distances between pairs of viruses based on gene presence-absence data and full-length DNApol sequences (Table 3) were highly correlated (Mantel test), whether chloroviruses were included in the analysis (*r* = 0.99), or not (*r* = 0.96). Pairs of viruses with a high degree of similarity in their gene content, measured by gene presence-absence, also have low phylogenetic distances based on full-length DNApol sequences. Amplicons from environmental samples have to be clustered at an appropriate identity level that is specific to the full length DNApol. Correlating the variation in phylogenetic distance of full length DNApol sequences to different levels of amino acid (aa) identity of corresponding amplicons showed decreasing variation with increasing stringency (Figure 5). The variation approached zero when clustering DNApol fragments at 97% identity, which was applied to the environmental sequences in this study.

### 3.3. Environmental Prevalence of Prasinoviruses Show Adaptation to Environmental Conditions

**Figure 6:**
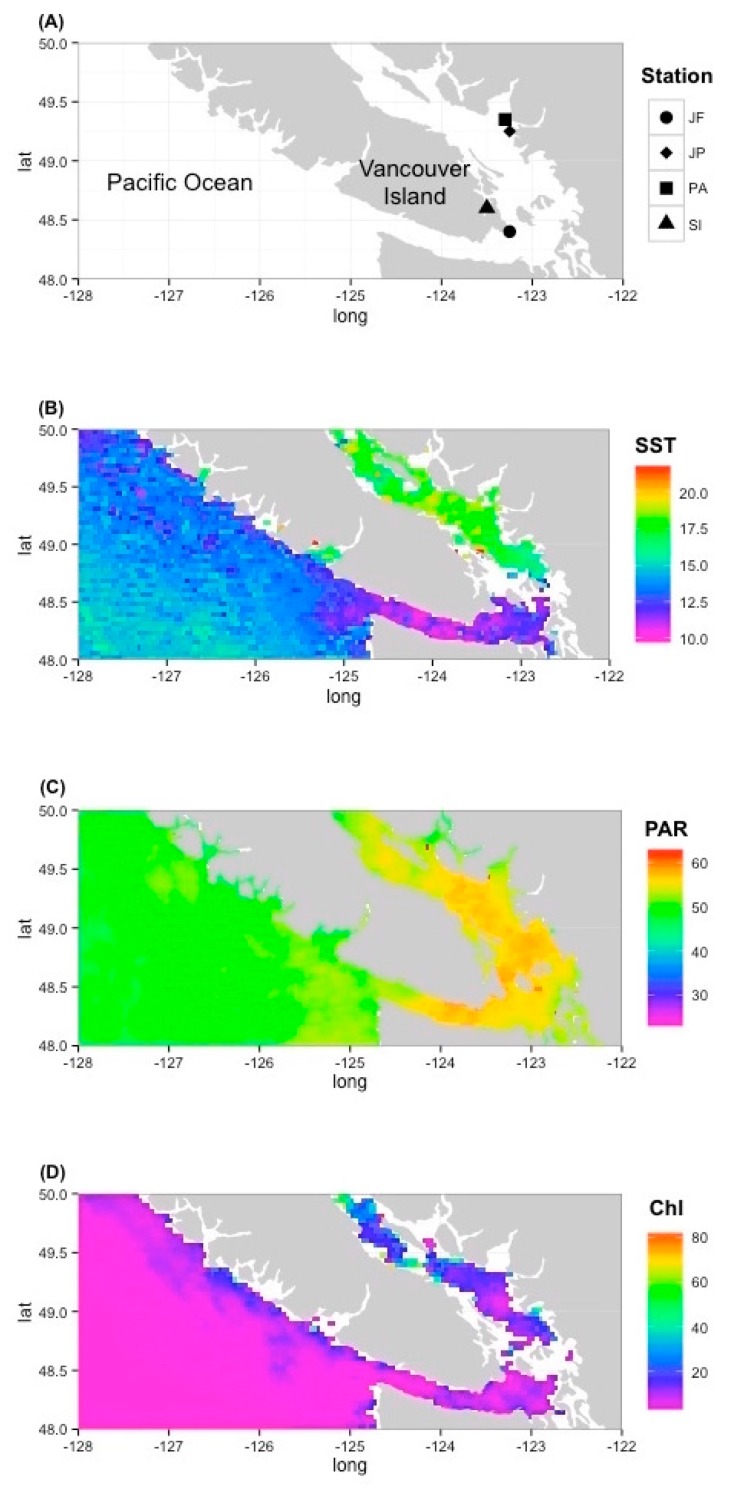
Sampling locations (**A**) and corresponding environmental parameters, sea surface temperature ((**B**) SST, °C), photosynthetically-active radiation ((**C**) PAR, μmol photons m^−2^ s^−1^) and Chlorophyll *a* concentration ((**D**) Chl, mg m^−3^), all based on 32-day composite data from the Aqua MODIS satellite. Sampling stations: Juan de Fuca Strait (JF), Jericho Pier (JP), Point Atkinson (PA), and Saanich Inlet (SI). lat: Degree latitude; long: Degree longitude.

To study the distribution of prasinovirus ecotypes in the five environmental samples, Jericho Pier (JP), Port Atkinson (PA), Juan de Fuca Strait (JF), Saanich Inlet surface layer, and Saanich Inlet deep layer (Figure 6) were compared. Samples from Saanich Inlet (SI) were collected at seven time points over the course of a year from 10 m (SI surface) and 200 m (SI deep), and were analyzed as annual composites from the surface layer and the deep layer at this site. Estimates from Aqua MODIS satellite data, averaged over 32 days around the sampling dates showed ambient SST of 18 °C, PAR of 45–50 μmol photons m^2^ s^−1^ and Chl *a* of 25–30 mg m^−3^ at JP and PA, while at JF the estimates were 10 °C SST, 55 μmol photons m^2^ s^−1^ PAR and 10 mg m^−3^ for Chl *a.* In situ salinity was 23 PSU for PA and JF and 12 PSU for JP. Temperature and salinity for the SI surface and deep samples, measured in situ, averaged 7.5 and 9.4 °C and 30 and 31 PSU (Supplementary Table S1).

**Figure 7:**
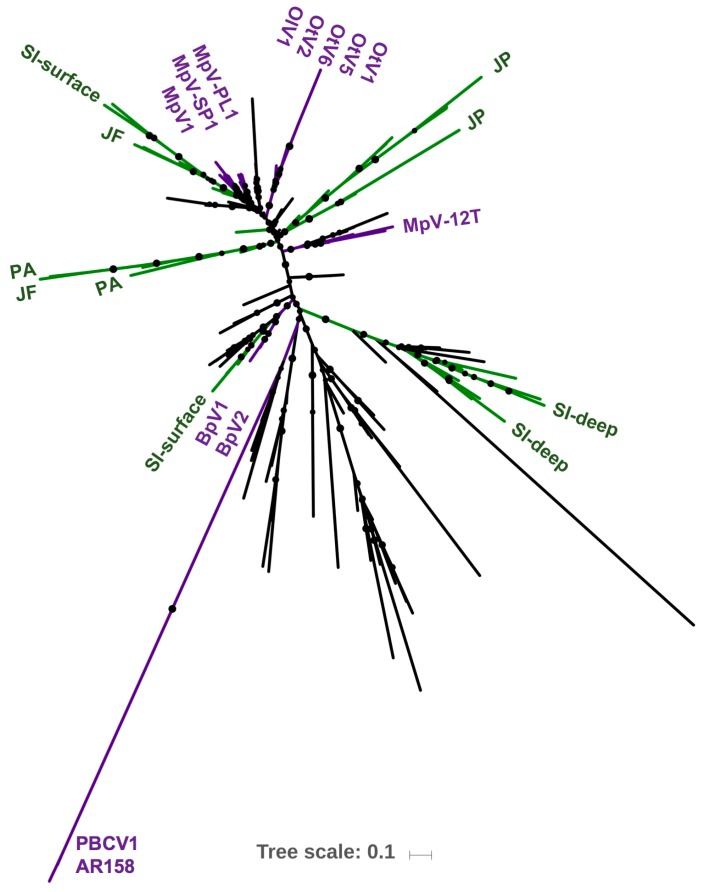
Maximum likelihood tree of 197 partial phycodnavirus DNApol sequences from five environmental samples clustered at 97% aa identity. Reference sequences are highlighted in purple, dominant OTUs and branches for the environmental samples are indicated in green for JP, PA, JF, SI. Black branches represent other operational taxonomic units (OTUs). Bootstrap values indicate branch support, values from 50 to 100% are shown as size-dependent circles.

Combined over all samples, environmental DNApol fragments from phycodnaviruses of about 129 aa length, pooled at 97% similarity, produced 197 operational taxonomic units (OTUs), including the references. Phylogenetic analysis of these sequences revealed that they clustered into several groups, with most nodes being supported by bootstrap values above 75% (Figure 7). The distribution of reference sequences on the tree matches the topology of trees based on gene presence or absence (Figure 3), and full-length DNApol sequences (Figure 4). Some of the environmental sequences groups were associated with sequences from prasinovirus isolates, while others were distant from known prasinovirus sequences. Moreover, the most abundant environmental sequence from each sample clustered relatively near a sequence from a prasinovirus, with the exception of the most abundant sequence from the SI deep sample, which lies on a distant branch that only contains environmental sequences. *Chlorella* viruses are on a distant branch, *Bathyococcus* viruses are clearly separated, and the *Ostreococcus* viruses are clustered together. The *Micromonas* viruses MpV-PL1, MpV-SP1, and MpV1 branch closely together with SI surface and JF sequences, while MpV-12T is distant from the other *Micromonas* viruses. Numerous branches of environmental sequences are not represented by sequences from isolates. For each of the five environmental samples the dominant OTUs were placed on distinctive branches of the phylogenetic tree. Dominant OTUs in JP and in SI-deep are represented on separate branches, while for PA and JF they overlap (Figure 7).

## 4. Discussion

This study highlights the similarities and differences among the genomes of *M. pusilla* viruses and other phycodnaviruses, as well as their distribution in the environment. In particular, the results show that there is substantial overlap in the gene content among viruses infecting the genera *Micromonas*, *Ostreococcus*, and *Bathyococcus*; however, there is also a large “flexible” component to their genomes. Moreover, there is considerable divergence among the *Micromonas* viruses, with the variation within these viruses being as large as it is among the sequenced prasinoviruses. Finally, an analysis of environmental DNApol sequences reveal an expansive diversity of viruses closely related to prasinovirus isolates and a niche-specific distribution of ecotypes in environmental samples. These findings are discussed in detail below.

### 4.1. Origin and Distribution of Genes in *Micromonas* Viruses

The *Micromonas* viruses MpV1, MpV-PL1, and MpV-SP1 show a high degree of genome similarity to each other, as well as to *Ostreococcus* viruses in terms of the number of ORFs, ORF length, GC content, and tRNAs, and in comparison to *Bathyococcus* viruses [11,12] and MpV-12T. Specifically, MpV-12T has a lower GC content and larger ORF length, and is more similar to *Bathyococcus* viruses, and was isolated on a different host strain than MpV-PL1 and MpV-SP1. Additionally, although MpV-12T has a wide host range [14], it does not infect the host of MpV-PL1 and MpV-SP1. Moreover, genomes were compared for homologues by clustering at an amino acid identity of 50%. This cut-off was selected based on identities among obvious homologues by annotation and based on the sensitivity of the UCLUST algorithm, which applies the same identity definition as BLAST [42]. MpV-PL1, MpV-SP1, and MpV1 shared most of their genes, while more than half of the MpV-12T genome was not shared with the other *Micromonas* viruses (Figure 1). Having only 80 genes shared among the *Micromonas* viruses at this identity level is low relative to the seven sequenced *Ostreococcus lucimarinus* viruses [11], which shared most of their genes and had pairwise nucleotide identities above 60% for their core genes.

The identification of 80 ORFs with functional annotation that were shared among all of the *Micromonas* viruses was the basis for defining a core genome among this group of viruses, with the rest of the genes being assigned as the “flexible” pan-genome. These high similarity core genes were supplemented with results from an additional BLAST-P analysis, which increased the total core genome to 119 putative genes. This additional analysis revealed genes for viral replication and virion structure, as well as *phoH*, a gene which is induced under phosphate stress (Table 2). *PhoH* is widely distributed in marine phage and has been used as an alternative marker gene [55,56] for phages and also eukaryote viruses in diversity studies, yet its exact function is not well defined. The core genes associated with viral replication were also found in *Ostreococcus* viruses, although the set of conserved genes in *Micromonas* viruses appears lower than described for *Ostreococcus* viruses [10,11,13]; however, it is much larger than that found in the NCLDV super group [8].

In contrast to the core genome, there is also a shared, but flexible, pan-genome that varies among *Micromonas* viruses. Most of these ORFs have no functional annotation, and those that do have been seen in other prasinovirus genomes. The gene complex for amino-acid synthesis found in MpV1 and *Bathyococcus* viruses [12] is also present in MpV-PL1. Both MpV-PL1 and MpV-12T carry a copy of heat shock protein 70 despite their otherwise limited genome overlap. Two transketolase genes, part of the Calvin cycle and pentose phosphate pathway, are only found in MpV-SP1, but have phycodnavirus homologues in metagenomes from Yellowstone Lake [57]. A homologue of 6-phosphofructokinase, a key enzyme of glycolysis, is present in MpV-PL1 and MpV-SP1, similar to *Ostreococcus* viruses [13]. Given that all these genes were expressed during a transcriptional study of *M. pusilla* UTEX LB991 infected by MpV-SP1 [58], similarly to the expression of transaldolase, glucose-6-phosphate dehydrogenase and 6-phospohgluconate dehydrogenase in cyanophages [59], these genes may influence the host’s metabolism during infection to boost viral replication. Furthermore the presence of dTDP-d-glucose 4,6-dehydratase and glycosyl transferase in MpV1, PL1, and SP1 indicates activity in sugar manipulation and potential glycosylation of proteins, similar to findings of glycosyl transferase in the *Ostreococcus* virus OtV1 [13]. As well, UDP-glucose 6-dehydrogenase found in MpV-12T and MpV SP1 could feed products of glycolysis into glycosylation of proteins of e.g., the capsid, similar to suggestions by Weynberg et al. [13] and Wang et al. [60]. Altogether, these presumably cell-derived metabolic genes have the potential to boost critical cell function for viral replication and could, thus, be beneficial to viral production.

The search for host homologues of viral genes resulted in only six ORFs being shared between *Micromonas* viruses and the host strain for MpV-PL1 and MpV-SP1 at a similarity level of 50%. In contrast, *Ostreococcus* viruses share 11 genes with their host [13], but often at lower amino acid identities to host homologs. A more detailed BLAST-P analysis of *Micromonas* virus ORFs that have a functional annotation revealed that most ORFs have the highest similarity to ORFs typically found in other viruses. Moreover, OTUs with high similarity to cellular sequences were from bacteria and eukaryotes that are not potential host taxa (Figure 2). This is similar to findings for the *Ostreococcus* virus OtV5 [10] and the Mollivirus, a NCLDV that infects *Acanthamoeba* [30]. Another comparison of prasinoviruses of different hosts also revealed a pattern of shared metabolic genes with an origin outside the host range, suggesting horizontal gene transfer [12]. Furthermore, horizontal gene transfer is believed to be the main mode to acquire novel genes for viruses of *Ostreococcus* and *Micromonas* [61], and to be beneficial for the virus [62]. Chlorovirus genomes show a similar pattern with a relatively large flexible pan-genome, a wide range of protein homologues, and evidence of horizontal gene transfer [63]. The data presented here provides putative evidence that horizontal gene transfer from a range of sources is widespread among viruses of *Micromonas*, possibly under selection pressure to adapt to environmental conditions.

### 4.2. Deriving Similarity in Gene Content from DNA Polymerase

Measuring the prevalence of virus with specific genetic repertoires, ecotypes, in the environment poses a challenge. This problem was approached by first constructing a phylogenetic tree based on the presence and absence of genes, in order to infer how closely related the viruses were to each other (Figure 3) and then set it in correlation to the phylogeny based on full-length DNApol sequences (Figure 4) and PCR amplicons.

The phylogeny based on gene presence and absence data presents an overall view of the genetic similarity among the prasinoviruses and its relationship to *Chlorella* viruses. While *Ostreococcus* and *Bathyococcus* viruses form well-defined groups, the *Micromonas* viruses are more scattered among the tree with MpV-12T on an isolated branch, suggesting substantial gene loss and transfer among these viruses. The relatively low bootstrap values in the gene presence and absence tree is similar to other phylogenies based on this technique [30,31]. This reflects that *M. pusilla* viruses generally share many genes, but MpV-PL1 shares more genes with OtV5, a virus that infects *Ostreococcus* sp. (125), than it does with MpV-12T (93) (Supplementary Figure S1). Furthermore, the phylogenetic tree based on the presence and absence of genes is similar in topology to the phylogenetic relationship inferred from whole-gene DNApol sequences, as well as others based on DNApol sequences or the presence and absence of genes [8,11,28,29,30].

Comparing pairwise phylogenetic distances based on gene presence and absence and DNApol showed strong congruency in the Mantel test (Table 3). This implies that DNApol sequences can be used to infer phylogenetic relationships among environmental sequences to assess the diversity of prasinoviruses in environmental samples as has been done [21,28], and that it is a strong proxy to infer the gene content among prasinoviruses.

However, because PCR only amplifies a gene fragment, sequences need to be clustered at 97% amino acid identity to be specific to full length DNApol sequences of viruses with diverse gene content. This is less stringent than Short and Short [23], who clustered the nucleotide level at 97% and Bellec et al. [35] who considered differences by single nucleotides as defining a distinct *Ostreococcus* virus haplotype. In contrast, it is more stringent than clustering at 75% identity, which was used in another study on prasinovirus distribution [36]. Overall, the identity level used in this study is appropriate to approximate the similarity and difference in gene content of viruses in environmental samples.

### 4.3. Environmental Prevalence of Prasinoviruses Show Adaptation to Environmental Conditions

With a framework to infer the phylogenetic relationship and similarity in the genetic repertoire among prasinoviruses based on DNApol amplicons, the approach was used to determine how well represented the sequenced prasinoviruses were across environmental samples. The four *Micromonas* viruses examined in this study were isolated from widely separated geographic areas. MpV-SP1 and MpV-PL1 were isolated from water collected from Scripps Pier (San Diego, CA, USA) and Port Aransas, (TX, USA), respectively [64], MpV1 was isolated from a eutrophic coastal lagoon in the northwestern Mediterranean [12], and MpV-12T was isolated off of the Dutch coast [14]. Although *Micromonas* viruses occur in the coastal waters of British Columbia [64,65], none of the sequenced isolates were from the region; hence, it was unknown if these genotypes would be well represented in these waters.

Five environmental samples from British Columbia coastal waters that reflect a range of conditions were analyzed for prasinovirus ecotypes and in situ conditions. Saanich Inlet is productive and stratified in spring and summer, and is isolated from deeper waters beyond the inlet because of a shallow sill; this leads to hypoxic deeper waters [66]. JP is strongly stratified, with fresh water influenced by water from English Bay that is adjacent to the city of Vancouver, while PA is more exposed and mixed with a higher salinity. JF is off the coast of Victoria in very exposed and mixed waters of the Juan de Fuca Strait [67]. This is described by the prevailing salinity, temperature, and chlorophyll concentrations at the sampling locations (Figure 6). While JP and PA are similar in their high SST of 18 °C and Chl *a* concentrations, JF is a much deeper mixed water body with a SST of only 10 °C and lower Chl *a* concentration. However, PA is more similar to JF in terms of salinity with both being 23 PSU. The combined DNApol sequences from all five environmental samples produced 197 distinct OTUs which were used to build a diverse and well-supported maximum likelihood DNApol tree displaying the prasinovirus and chlorovirus diversity.

The multitude of well-defined branches on the DNApol tree suggest a great diversity in prasinovirus ecotypes and their genetic repertoires, and visualizes their distribution across environments (Figure 7). The distribution of reference viruses on the tree generally reflects the tree topology of the reference tree-based full-length DNApol sequences and the presence and absence of genes, confirming the approach. The environmental OTUs substantially increases the known richness of prasinoviruses, and especially *Micromonas* viruses, in the environment. Furthermore, the specific distribution of the representative OTUs for each of the five environments suggests a specialization of the corresponding viral ecotypes to prevailing conditions. The Saanich Inlet samples, being long-term integrated samples, should rather be seen in comparison to each other than to the other three samples. Despite the Aqua MODIS data showing JP and PA being similar in temperature and JP, PA, and JF having similar Chl *a* concentrations and PAR levels, PA and JF are more similar environments based on their in situ salinities and presumed mixing. This is also reflected in the dominant prasinovirus genotypes for the samples. Saanich Inlet deep sequences and the stratified, near-shore JP sequences are on separate isolated branches. The dominant sequences in Saanich Inlet surface samples, and especially the two mixed, more saline PA and JF samples, share branches. This specialization of viruses to environments is congruent with findings that prasinovirus communities in the Northwest Mediterranean Sea are affected by environmental variables, and especially nutrient availability [36]. Additionally, considering the relatively wide host range of these viruses within a genus [14,62] the pattern likely represents a response by the prasinovirus community to the specific environmental conditions and not solely the host community. Altogether this could mean that prasinovirus ecotypes with similar genetic repertoires, approximated by DNApol similarity, dominate in similar environments.

In conclusion, this research highlights the genetic repertoire encoded by prasinoviruses infecting *M. pusilla* and other prymnesiophytes. We identified a core set of genes that are shared among *Micromonas* viruses despite their marked differences, and identified a large set of genes that make up a flexible part of the genome, implying that there is a large “pan-genome” that is shared among prasinoviruses. Furthermore, we set the *Mircomonas* virus genomes in contrast to genomes from other prasinoviruses, phycodnaviruses, and a host genome elucidating the overlap in gene content. The presumed origin of shared genes and their distribution across viral clades shows a complex evolutionary history and horizontal gene transfer. The diversity in prasinovirus genomes is linked to their distribution pattern in nature, implying adaptation of viral ecotypes to their environment.

## Figures and Tables

**Table 1:** General genome characteristics of *Micromonas pusilla* (MpV)-PL1 and MpV-SP1 compared to MpV1 and MpV-12T.

	MpV-PL1	MpV-SP1	MpV1	MpV-12T
Genome size (bp)	196,960	173,350	184,095	205,622
Host	*Mp*UTEX991	*Mp*UTEX991	*Mp*RCC1109	*Mp*LAC38
# ORF	275	248	244	253
ORF length	684	659	715	749
% GC	43.3	40.6	41.0	39.8
Asn-tRNA	1	1	1	2
Gle-tRNA	1	1	1	1
Ile-tRNA	1	1	1	1
Leu-tRNA	0	1	1	1
Thr-tRNA	1	1	1	1
Tyr-tRNA	1	1	1	1

Host refers to original host of isolation; open reading frame (ORF) length is average; % GC for whole genomes; transfer RNAs (tRNAs) present in genomes and their number of copies.

**Table 2:** Examples of the core and pan-genome of four *M. pusilla* viruses after a combined cluster and Protein BLAST (BLAST-P) analysis.

Core-Genes		Pan-Genes	
Class	Putative Function	Class	Putative Function
DNA replication	DNA polymerase	AA synthesis	Acetolacetate synthase
	DNA topoisomerase		Acetolactate synthase
	DNA ligase		Aminotransferase
	DNA primase	DNA repair	Heat shock protein 70
Nucleotide metabolism	RNase/		Site specific DNA methylases/
	Ribonuclease		methyltransferases
	Ribonucleotide reductase		
Transcription	mRNA capping enzyme	Sugar manipulation	dTDP-d-glucose 4,6-dehydratase
	Transcription initiation factor		UDP-glucose 6-dehydrogenase
	Transcription elongation factor		6-phosphofructokinase
Structural genes	Capsid protein		Transketolase N-terminal
	Major capsid protein		Transketolase B subunit
Metabolism	PhoH	Total Shared	108
Total Core	119	Total Unique	327

The annotation is based on MpV-PL1; AA: Amino Acid; PhoH: Phosphate starvation-inducible protein.

**Table 3:** Phylogenetic distance between pairs of reference viruses based on full length DNApol and gene presence or absence.

		Presence-Absence (aa id 50%)												
	CDS Clusters	MpV1	MPV-12T	MpV-PL1	MpV-SP1	BpV1	BpV2	OtV1	OtV2	OtV5	OtV6	OlV1	PBCV1	AR158
DNApol	MpV1	**244**	0.98	0.64	0.66	1.11	1.08	0.63	0.72	0.69	0.57	0.68	4.99	5.40
	MPV-12T	0.36	**252**	1.02	0.99	1.18	1.16	1.06	1.05	1.11	1.00	1.06	5.41	5.01
	MpV-PL1	0.26	0.36	**271**	0.30	1.12	1.10	0.71	0.75	0.75	0.67	0.73	5.04	5.45
	MpV-SP1	0.23	0.36	0.18	**244**	1.09	1.10	0.70	0.75	0.77	0.69	0.71	4.99	5.40
	BpV1	0.38	0.42	0.38	0.39	**202**	0.24	1.07	1.16	1.17	1.15	1.13	4.89	4.90
	BpV2	0.39	0.42	0.38	0.39	0.05	**209**	1.05	1.17	1.13	1.11	1.13	4.91	4.92
	OtV1	0.27	0.33	0.24	0.25	0.37	0.38	**230**	0.29	0.25	0.42	0.24	5.36	5.37
	OtV2	0.26	0.33	0.23	0.24	0.41	0.41	0.05	**235**	0.33	0.48	0.22	6.07	6.08
	OtV5	0.27	0.33	0.25	0.25	0.37	0.38	0.01	0.06	**260**	0.46	0.28	5.42	5.43
	OtV6	0.26	0.35	0.25	0.24	0.38	0.38	0.09	0.10	0.09	**249**	0.45	5.40	5.41
	OlV1	0.28	0.35	0.25	0.24	0.39	0.40	0.08	0.08	0.08	0.09	**246**	6.09	6.10
	PBCV1	2.09	2.15	2.19	2.05	2.03	2.05	2.21	2.21	2.22	2.13	2.16	**789**	0.81
	AR158	2.15	2.18	2.23	2.10	2.07	2.08	2.27	2.27	2.30	2.22	2.25	0.12	**806**
	Mantel Test	0.96, *p* = 0.01										0.99, *p* = 0.01		

Bold numbers are the number of CDS compared per genome. The Mantel test was performed between the two distance matrices for all viruses, excluding *Chlorella* viruses.

## Supplementary Materials

The following are available online at www.mdpi.com/1999-4915/9/5/116/s1, Figure S1: Venn diagram of the CDS of four prasinoviruses, based on clusters by 0.5 amino-acid identity, Table S1: Sampling details and in situ conditions for environmental amplicon sequences from Jericho Pier, Point Atkinson, Juan de Fuca Strait, and Saanich Inlet.
