# Supplementary material for: Variation in the Genetic Repertoire of Viruses Infecting Micromonas pusilla Reflects Horizontal Gene Transfer and Links to Their Environmental Distribution
Source: Viruses. 2017 May 19;9(5):116. doi: 10.3390/v9050116 (PMC5454428; doi:10.3390/v9050116)
Supplement: Supplementary file 1 [file viruses-09-00116-s001.pdf]

Supplementary Materials: Variation in the Genetic Repertoire of Viruses Infecting Micromonas pusilla Reflects Horizontal Gene Transfer and Links to Their Environmental Distribution

Jan F. Finke, Danielle M. Winget, Amy M. Chan and Curtis A. Suttle

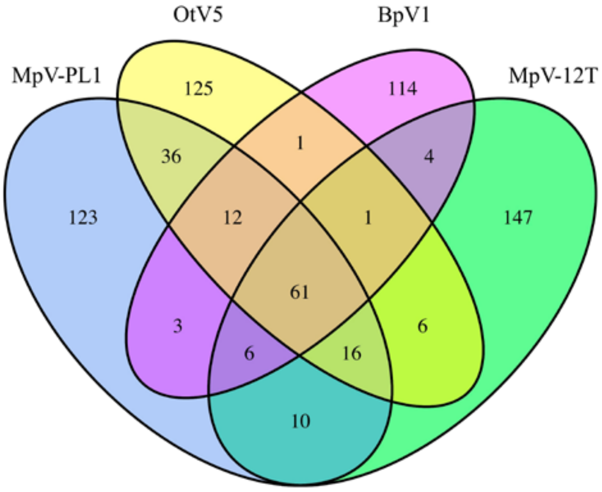

Figure S1. Venn diagram of the CDS of four prasinoviruses, based on clusters by 0.5 amino-acid identity.

Table S1. Sampling details and in situ conditions for environmental amplicon sequences from Jericho Pier, Point Atkinson, Juan de Fuca Strait and Saanich Inlet. Sampling date, location and depth given with in situ temperature (Temp.), salinity (Sal.). Field sampling volume (Vol.), type of pre-filter used in the lab, volume of viral concentrate (VC) and volume used for DNA extraction (Ext.) used are stated.

Site	Year	Month	Lat, Long	Depth (m)	Temp. (°C)	Sal. (PSU)	Vol. (L)	Pre-filter	VC (ml)	Ext. (ml)
Jericho Pier (JP)	2006	June			18	12	41		900	650
Point Atkinson (PA)	2006	June	48.45°N, 123.32°W	1	14	23	45	GC50-HVLP	795	650
Juan de Fuca Strait (JF)	2006	July			8.3	33	72		620	420
	2007	April			na	na	~20		245	1.7
	2008	February			7.5	31	18		287	7.8
	2008	March			7.5	31	18		168	5.9
	2008	April		10	7.6	31	16		230	2.9
	2008	June			9.8	33	14		185	1.1
	2008	August			12	34	18		190	3.1
Saanich Inlet (SI)	2008	December	48.58°N, 123.5°W		9	32	15	GF/D-Sterivex	210	11
	2007	April			na	na	17		220	10
	2008	February			9.4	34	17		300	8.9
	2008	March			9.4	34	16		220	23
	2008	April		200	9.4	34	19		250	8.3
	2008	June			9.4	34	18		235	15
	2008	August			9.4	34	19		160	78
	2008	December			9.2	34	16		186	41

© 2017 by the authors. Submitted for possible open access publication under the terms and conditions of the Creative Commons Attribution (CC BY) license (http://creativecommons.org/licenses/by/4.0/).
